# Anonymous Three-Party Password-Authenticated Key Exchange Scheme for Telecare Medical Information Systems

**DOI:** 10.1371/journal.pone.0102747

**Published:** 2014-07-21

**Authors:** Qi Xie, Bin Hu, Na Dong, Duncan S. Wong

**Affiliations:** 1 Hangzhou Key Laboratory of Cryptography and Network Security, Hangzhou Normal University, Hangzhou, China; 2 Department of Computer Science, City University of Hong Kong, Kowloon, Hong Kong, China; King Saud University, Kingdom of Saudi Arabia, Saudi Arabia

## Abstract

Telecare Medical Information Systems (TMIS) provide an effective way to enhance the medical process between doctors, nurses and patients. For enhancing the security and privacy of TMIS, it is important while challenging to enhance the TMIS so that a patient and a doctor can perform mutual authentication and session key establishment using a third-party medical server while the privacy of the patient can be ensured. In this paper, we propose an anonymous three-party password-authenticated key exchange (3PAKE) protocol for TMIS. The protocol is based on the efficient elliptic curve cryptosystem. For security, we apply the pi calculus based formal verification tool ProVerif to show that our 3PAKE protocol for TMIS can provide anonymity for patient and doctor while at the same time achieves mutual authentication and session key security. The proposed scheme is secure and efficient, and can be used in TMIS.

## Introduction

In the traditional medical diagnosis process, a patient goes to a hospital or clinic, and then consults a doctor. With the advancement of computer and network technologies, many countries and regions are establishing telecare medical information systems (TMIS), for making the medical diagnosis process more efficient, reliable and effective. With TMIS, patients can save time and have access to doctors and specialists more easily. Furthermore, patient records can also be exchanged between various hospitals and clinics. The system is also providing enhanced efficiency and effectiveness, especially on doing some basic diagnoses at patients' home [1]. Furthermore, TMIS is also useful for cases where chronic patients are involved. For example, through TMIS, a hypertension patient or a diabetes mellitus patient could exchange his/her daily medical data collected by the patient at home and the medical advice from doctors or nurses directly without requiring the patient to pay a visit to a hospital or a clinic. For emergency patients, such those with angina pectoris, hyperpyretic convulsion and asthma attacks, the TMIS can help exchange the medical records of a patient in concern, for example, between the database of a family doctor and the ICU of a hospital.

In TMIS, patients, doctors and nurses can register onto a trusted medical server (TS) and use passwords to perform authentication or secure channel establishment with the TS. Once a patient needs to consult a doctor, the patient can contact a doctor, and communicate with the doctor through a secure communication channel. For achieving these objectives, anonymous three-party password-authenticated key exchange (3PAKE) protocols for TMSI should be addressed. The 3PAKE protocol is to achieve mutual authentication between a patient and a doctor with the aid of the TS, and at the same time, ensure that an adversary does not know the exact identities of both the doctor and the patient. Furthermore, 3PAKE helps establish a secure channel via generating jointly a session key, which is then used for building a secure channel between the patient and the doctor.

In 2007, Lu and Cao [2] proposed an efficient 3PAKE scheme. However, Guo et al. [3], Chung and Ku [4], Phan et al. [5] and Nam et al. [6] later showed that Lu and Cao's scheme is vulnerable to undetectable on-line dictionary attack, off-line password guessing attack, and man-in-the-middle attack, respectively. In 2009, Huang [7] proposed another 3PAKE scheme, which was later shown by Yoon and Yoo [8] that it cannot defend against undetectable password guessing attack and off-line password guessing attack. In 2011, Lou and Huang [9] proposed a new 3PAKE scheme. The scheme is based on Elliptic Curve Cryptosystem (ECC) and is efficient. However, Xie et al. [10] recently showed that Lou and Huang's scheme is vulnerable to off-line password guessing attack and partition attack. Xie et al. also proposed an improved scheme for solving these problems. In 2012, Yang and Cao [11] and Chen et al. [12] also proposed modular exponentiation based and ECC-based 3PAKE schemes, respectively. However, these schemes, when compared with other existing schemes, require heavier computation costs. In 2010, Wang and Zhao [13] proposed a three-party key agreement protocol based on chaotic maps. Later, Yoon and Jeon [14] showed that their scheme is vulnerable to illegal message modification attack, and then proposed an improved one. Unfortunately, both schemes require a reliable third party, which shares a different long-term cryptographic key with each participant, it is inconvenient that each participant should protect the long-term secret key. Furthermore, these schemes are not as efficient as previous 3PAKE schemes. In 2013, Xie et al. [15] proposed the first chaotic maps-based 3PAKE scheme without using timestamp.

In light of all the schemes mentioned above, we notice that none of them can support privacy protection, since anyone can obtain user's identity from the authentication process. As we know, user's privacy protection is very important in some applications, such as telecare medical information systems (TMIS). In 2012, Lai et al. [16] proposed a smart-card-based anonymous 3PAKE using extended chaotic maps. However, Zhao et al. [17] showed that the scheme is vulnerable to the privileged insider attack and the off-line password guessing attack, and proposed an improved one. In 2013, Lee et al. [18] proposed another anonymous 3PAKE scheme using Chebyshev chaotic maps, but their scheme is suffering from the man-in-the-middle attack once after an attacker gets the identity of each participant, which in practice is easy to obtain.

Based on the advantages of elliptic curve cryptosystem (ECC), that is, having shorter secret keys and faster computational speed, it is desirable if an ECC-based anonymous 3PAKE scheme can be built for TMIS. To the best of our knowledge, however, there is no ECC-based anonymous 3PAKE scheme is proposed. In this paper, we propose the first ECC-based anonymous 3PAKE scheme, and show that it is efficient.

The rest of the paper is organized as follows. In Section 2, we propose an anonymous 3PAKE scheme. The security analysis of the scheme is given in Section 3. After that, other security discussions and the performance comparison are described in Sections 4. The paper is concluded in Section 5.

## The Proposed Scheme

In this section, we propose an anonymous 3PAKE scheme. Some notations will be used in this paper are defined as follows.

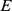
: an elliptic curve defined over a finite field with large order 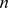.

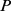
: a generator on *E* with large order 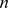.

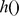
: a secure one-way hash function which maps to an integer.

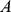
: user 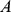, may be a patient.

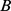
: user 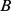, may be a doctor or nurse.

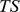
: trusted medical sever.

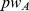
: user
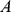
's password, shared with 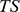.

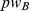
: user
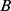
's password, shared with 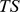.
*ID_A_*, *ID_B_*, *ID_TS_*: identities of 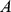, 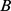 and 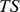, respectively.

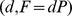
: *TS*'s private-public key pair.

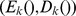
: secure symmetric encryption/decryption functions with key 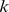.

The proposed anonymous 3PAKE scheme is described as follows. Algorithm 1 illustrates the proposed scheme.

**Step 1**: User *A* randomly chooses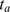, and computes 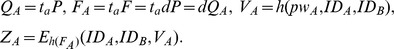
Then sends 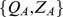 to 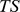.

**Step 2**: Upon receiving 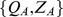, the trusted server *TS* computes 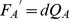, and decrypts 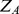 to obtain 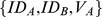, computes 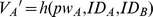 and verifies if 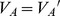. If not, terminates. Otherwise, user *A* is authenticated. Thus, *TS* knows that user *A* wants to establish a shared session key and communicate with a user *B*. *TS* randomly chooses an integer 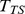, computes 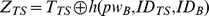, and sends 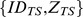 to *B*.

**Step 3**: Upon receiving 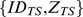, user *B* computes 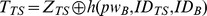 and randomly chooses 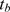, computes



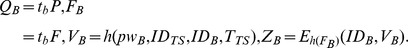
Then sends 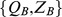 to *TS*.

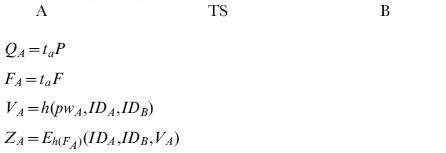


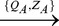


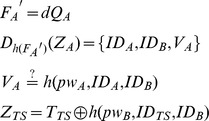


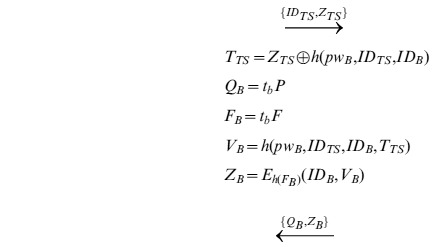


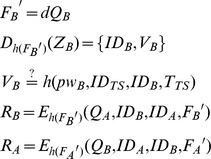


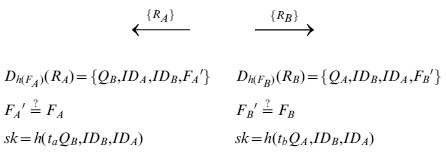


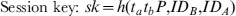

            Algorithm 1 The proposed anonymous 3PAKE scheme

**Step 4**: Upon receiving 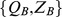, *TS* computes 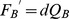, and decrypts 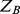 to obtain 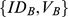. Then *TS* computes 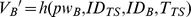 and verifies if the decrypted
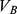
is correct or not by 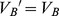. If not, terminates. Otherwise, user *B* is authenticated.

*TS* computes and sends 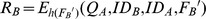 to *B*, computes and sends 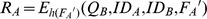 to *A*.

**Step 5**: Upon receiving 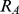 or 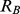 from *TS*, *A* decrypts 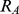 and gets 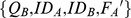. Then *A* checks the validity of 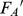, and computes 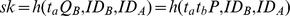 as the session key. At the same time, *B* decrypts 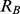, and gets 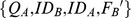. After checking the validity of 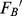, *B* computes 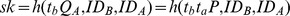 as the session key shared with *A*.

## Security Analysis

In this section, we use applied pi calculus [19] based formal verification tool ProVerif [20] to show that the proposed scheme satisfies anonymity, authentication and security. ProVerif is an automatic cryptographic protocol verifier in the formal model and supports automatic and effective security analysis of many cryptographic primitives such as symmetric and asymmetric encryption, digital signature, hash function, Diffie-Hellman key agreements, etc [21].

### 3.1 Authentication and security

We model the protocol steps according to the message sequences shown in section 2. In particular, public channel ch1 is used for the communication between user *A* and the trusted medical server *TS*, and public channel ch2 is used for the communication between user *B* and *TS*.

(* -------------channel--------------------*)
ch1: communication channel between A and TS
ch2: communication channel between B and TS
free ch1: channel.
free ch2: channel.

We then define two variables SKA and SKB, which are the session keys calculated by *A* and *B*, respectively.

(* -------------shared keys --------------------*)
free SKA: bitstring [private].
free SKB: bitstring [private].

The constants IDA, IDB and IDTS denote the identities of *A*, *B*, and *TS*, and PWA and PWB denote the passwords of *A* and *B* shared with *TS*, respectively. Let d be *TS*'s secret key, and the constant P is the base point of group E.

(*--------------- constants and variables-----------------*)
free SKA: bitstring [private].
free SKB: bitstring [private].
const IDA: bitstring.
const IDB: bitstring.
const IDTS: bitstring.
const PWA: bitstring [private].
const PWB: bitstring [private].
const P: bitstring.
free d: bitstring [private].

The ProVerif code for non-logical constants and the corresponding equational theory is giving below:

(*---------------constructor----------------*)
fun h(bitstring): bitstring.    //*hash function
fun senc(bitstring, bitstring): bitstring.    //*symmetric encryption
fun xor(bitstring, bitstring): bitstring.
fun mult(bitstring, bitstring): bitstring.
(*---------------destructors & equations----------------*)
reduc forall x: bitstring, y: bitstring; sdec(senc(x, y), y)  = x.
equation forall x: bitstring, y: bitstring; xor(xor(x, y), y)  = x.

The core message sequences for the proposed scheme are given below. QA, ZA, ZTS, QB, ZB, RA and RB in these messages are computed by corresponding senders before they are transmitted.

(*---------------messages---------------*)
Message 1:A→TS: {QA, ZA}
   Message 2:TS→B: {IDTS, ZTS}
Message 3:B→TS: {QB, ZB}
   Message 4:TS→A,B: {RA},{RB}

The proposed protocol consists of the parallel execution of three processes: the user *A*, UserA, the trusted server TrustSever and another user *B*, UserB. The processes are the core of protocol model, which define the behavior of each participant in applied pi calculus. The process UserA defines the behavior of user *A*, who computes QA, FA, VA and ZA, and sends message (QA, ZA) through a public channel. After that, user *A* receives message RA and computes SKA. The process of UserA is modeled as below:

   (*-------------------UserA's process-------------------*)
let UserA  = 
   new ta: bitstring;
   event UserStarted(IDA);
   let QA  =  mult(ta,P) in
   let FA  =  mult(d,QA) in
   let VA  =  h(((PWA,IDA,IDB))) in
   let ZA  =  senc((((IDA,IDB,VA))),h(FA)) in
   out(ch1,(QA,ZA));
   in (ch1,RA': bitstring);
let (QB':bitstring,IDA'':bitstring,IDB'':bitstring,FA'':bitstring) = sdec(RA',h(FA)) in
   if FA'' = FA then
   let SKA = h(((mult(ta,QB'),IDB,IDA))) in
   0.

The process TrustSever defines the behavior of TS during authentication, it computes FA' and ZTS, and sends message (IDTS, ZTS) to UserB through a public channel2 when it receives message (QA, ZA) through a public channel1. After that, TrustSever receives message (QB, ZB), computes RA and RB, and sends RA and RB to UserA and UserB through public channel1 and channel2, respectively. The process of TrustSever is modeled as follows.

   (*-------------------TrustSever's process-------------------*)
let TrustSever  = 
   in(ch1, (QA':bitstring, ZA':bitstring));
   let FA' =  mult(d,QA') in
   let (IDA': bitstring,IDB': bitstring,VA': bitstring) =  sdec(ZA',th(FA')) in
   let VA'' = h(((PWA,IDA',IDB'))) in
   if VA' = VA'' then
   new TTS: bitstring;
   let ZTS = xor(TTS,h(((PWB,IDTS,IDB)))) in
   out (ch2,(IDTS,ZTS));
   in (ch2,(QB': bitstring,ZB': bitstring));
   let FB' = mult(d,QB') in
   let (IDB'': bitstring,VB': bitstring) = sdec(ZB',h(FB')) in
   let VB'' = h((((PWB,IDTS,IDB,TTS)))) in
   if VB' = VB'' then
   let RB = senc(((((QA',IDB,IDA,FB')))),h(FB')) in
   let RA = senc(((((QB',IDA,IDB,FA')))),h(FA')) in
   out(ch1,RA);
   out(ch2,RB).

The process UserB defines the behavior of user *B* during authentication, who computes TTS', QB, FB, VB and ZB, and sends message (QB, ZB) back to *TS* through a public channe2. After that, user *B* receives message RB and compute SKB. The process of UserB is modeled as follows:

   (*-------------------UserB's process-------------------*)
let UserB  = 
   in(ch2, (IDTS': bitstring, ZTS': bitstring));
   let TTS' = xor(ZTS',h(((PWB,IDTS',IDB)))) in
   new tb: bitstring;
   let QB = mult(tb,P) in
   let FB = mult(d,QB) in
   let VB = h((((PWB,IDTS',IDB,TTS')))) in
   let ZB = senc(((IDB,VB)),h(FB)) in
   out(ch2,(QB,ZB));
   in (ch2,RB': bitstring);
let (QA':bitstring,IDB'':bitstring,IDA'':bitstring,FB'':bitstring) = sdec(RB',h(FB)) in
   if FB'' = FB then
   event UserAuthed(IDA'');
   let SKB = h(((mult(tb,QA'),IDB,IDA))) in
   0.

The protocol is modeled as the parallel execution of the above three processes:

   process !UserA | !TrustSever | !UserB

The session key security is formalized by the following two queries for checking by Proverif:

(*--------------------query--------------------------*)
   query attacker(SKA).
   query attacker(SKB).

The Authentication of the protocol was modeled as a correspondence relation between two events: UserStarted and UserAuthed, which are inserted into the processes of UserA and UserB, respectively:

   event UserAuthed(bitstring).
   event UserStarted(bitstring).
query id: bitstring; inj-event(UserAuthed(id))  =  = > inj-event(UserStarted(id)).

We perform the above process in the latest version 1.85 of ProVerif and the performance results show that (1) the session key in the proposed scheme is secure under Dolev-Yao model; and (2) the authentication property is satisfied.

### 3.2 Anonymity

In ProVerif, strong anonymity is defined as follows [22].

Let 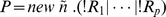 be a *p*-party protocol in its canonical form where 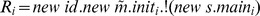 for any 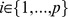. 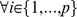, we build the protocol 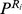 as:


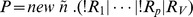
, where 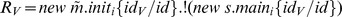.

The identity *id_V_* of the agent playing role *R_V_* is a public name, not under any new restriction in *P*. *P* is said to preserve strong anonymity of *R_i_* if 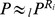. Informally, this means that the adversary cannot distinguish a situation where the role *R_V_* with known identity *id_V_* was executed from one in which it was not executed at all [23]. Going back to our proposed protocol, strong anonymity requires a system in which a user (*A* or *B*) with publicly known identity IDV executes the protocol to be indistinguishable from a system in which it is not present at all. We formally define user *A* and user *B* as follows:

let UserA  = 
   in(kc, xPKTS: bitstring);
   !(new ta: bitstring;
   let QA  =  mult(ta, P) in
   let FA  =  mult(ta, xPKTS) in
   let VA  =  h((pwa, IDA, IDB)) in
   let ZA  =  senc(h(FA), (IDA, IDB, VA)) in
   out(c, (QA, ZA));
   in(c, xRA: bitstring)).
let UserB  = 
   in(kc, xPKTS: bitstring);
   !(new tb: bitstring;
   in(c, (xIDTS: bitstring, xZTS: bitstring));
   let xTTS  =  sdecr(h((pwb, IDTS, IDB)), xZTS) in
   let QB  =  mult(tb, P) in
   let FB  =  mult(tb, xPKTS) in
   let VB  =  h((pwb, IDTS, IDB, xTTS)) in
   let ZB  =  senc(h(FB), (IDB, VB)) in
   out(c, (QB, ZB));
   in(c, xRB: bitstring)).

And formally define *TS* as follows:

let TS  = 
   new d:bitstring;
   !(let F  =  mult(d, P) in out(kc, F))
   |
   !(
   new TTS: bitstring;
   new rand: bitstring;
   in(c, (xQA: bitstring, xZA: bitstring));
   let FA  =  mult(d, xQA) in
   let (xIDA: bitstring, xIDB: bitstring, xVA: bitstring)  = 
   sdec(h(FA), xZA) in
   let VA  =  h((pwa, IDA, IDB)) in
   if VA  =  xVA then
   let ZTS  =  sencr(h((pwb, IDTS, IDB)), rand, TTS) in
   out(c, (IDTS, ZTS));
   in(c, (xQB: bitstring, xZB: bitstring));
   let FB  =  mult(d, xQB) in
   let (xIDB: bitstring, xVB: bitstring)  = 
   sdec(h(FB), xZB) in
   let VB  =  h((pwb, IDTS, IDB, TTS)) in
   if VB  =  xVB then
   let RB  =  senc(h(FB), (xQA, IDB, IDA, FB)) in
   let RA  =  senc(h(FA), (xQB, IDA, IDB, FA)) in
   out(c, RB);
   out(c, RA)
   ).

For verification, we use randomized symmetric encryption to conceal the random integer 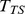 instead of using the exclusive-or. The proposed protocol is formally defined as:

   process !((UserA) | (UserB) | (TS))

Anonymity of users *A* and *B* is proved separately as follows. In order to show *A*'s anonymity, the proposed protocol is required to be observational equivalent to the augmented protocol defined as follows:

process !((UserA) | (UserB) | (TS)) |
   let IDA  =  IDV in ((UserA) | (UserB) | (TS))

The observational equivalence can be translated into the following ProVerif bi-process:

process !((UserA) | (UserB) | (TS)) |
   new ID: bitstring;
   let IDA  =  choice[ID, IDV] in ((UserA) | (UserB) | (TS))

The right hand side of the choice represents a system where a user with public identity IDV can run the protocol. The proposed protocol is simulated using the latest version 1.85 of ProVerif and simulation outcome shows that the scheme achieves the anonymity for user *A*. The anonymity of user *B* can be simulated and shown in a similar way.

## Security Discussions and Performance Comparison

In this section, we discuss some other aspects related to security, and then evaluate the performance of the scheme.

### 4.1 Discussions

#### 4.1.1 Offline password guessing attack

Suppose an adversary eavesdrops the communication between *A*, *B* and *TS*, and gets all the transmitted messages 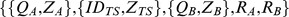. To launch the off-line password guessing attack, the adversary may choose a trial password 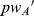 and compute 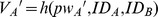. Even if the adversary knows 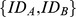, the adversary still cannot compute 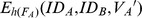 and therefore, cannot verify if 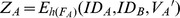 since the adversary does not know 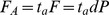 from 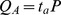 or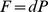 due to the intractability of the Computational Diffie-Hellman (CDH) problem. Therefore, the adversary cannot verify if his guessed 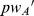
is correct or not.

If the adversary guesses *B*'s password 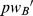, and computes 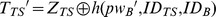, 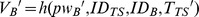, the adversary still cannot verify if 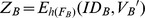 without knowing 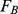. That is, the adversary cannot determine if his guessed 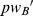 is correct or not.

Therefore, the proposed scheme can resist off-line password guessing attack. If an adversary launches on-line password guessing attack, *TS* may detect the attack since it needs to verify the correctness of 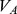 and 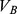.

#### 4.1.2 Perfect forward secrecy

In the proposed scheme, the session key is 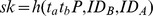, where 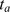 and 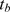 are nonces chosen by user *A* and user *B*, respectively. Even if an adversary can get *TS*'s secret key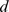, *A* and *B*'s passwords and identities, the adversary cannot compute the previous established session key due to the intractability of CDH problem.

#### 4.1.3 Replay attack

Suppose that an adversary impersonates *A* and replays *A*'s message 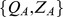 to *TS*, the adversary cannot compute 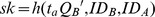 without knowing 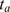. On the other hand, if an adversary impersonates *B* and replays *B*'s message 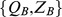 to *TS*, 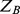 cannot pass the authentication checking by *TS* as 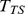
is a new nonce chosen by *TS* in each new session. The same reason applies if an adversary replays *TS*'s message 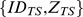,
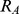
and 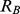. The replayed message cannot pass the verification performed by *A* and *B*, as 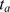
and 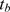
are new nonces chosen by *A* and *B*, respectively, and 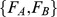 are refreshed in each new session.

#### 4.1.4 Forgery attack and impersonation

In our scheme, if an adversary attempts to impersonate *A* (or *B*, or *TS*) and sends messages to *TS* (or *B*, or *A*), but these messages cannot pass the verification process of *TS* (or *B*, or *A*) as the adversary does not know the password or secret key *d*.

#### 4.1.5 Man-in-the-middle attack

If an adversary attempts to launch the man-in-the-middle attack, the adversary has to generate and send the forgery messages to *TS* and has to pass the verification performed by the *TS*, before the adversary can obtain the session key shared with *A* and another session key shared with *B*. However, it is infeasible as the adversary does not know *d* or 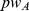 or 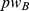.

### 4.2 Performance Analysis

Let 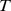, *D*, 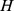 and 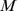 be the time for performing a Chebyshev polynomial computation, a symmetric encryption/decryption, a one-way hash function, and a scalar multiplication on elliptic curve, respectively. Li et al. [24] and Li et al. [25] showed that it needs 0.0005 second for completing one hash operation, 0.0087 second for one symmetric encryption/decryption, and 0.063075 second for one elliptic curve scalar multiplication operation, respectively. Kocarev and Lian [26] showed that it needs 0.07 second for a Chebyshev polynomial computation. As we know, these computation costs may vary due to different computational configurations and settings. However, in general, the elliptic curve scalar multiplication operation and the Chebyshev polynomial evaluation are slower than a symmetric key based encryption/decryption or a one-way hash function operation. The performance comparison between the scheme proposed in this paper and three other recently proposed ones [16]–[18] is given in Table 1.

**Table 1:** Performance comparison.

Schemes	User A	User B	Server	Total	Rounds	Estimated Time (s)
Lai et al. [16]	3T+6H	3T+6H	2T+8H+2D	8T+20H+2D	5	0.5847
Zhao et al. [17]	3T+6H+1D	3T+5H+1D	2T+8H+2D	8T+19H+4D	5	0.6043
Lee et al. [18]	3T+4H	3T +5H	2T +7H	8T +16H	4	0.568
Our scheme	3M+4H+2D	3M+5H+2D	2M+7H+4D	8M+16H+8D	4	0.5822

From Table 1, we can see that all schemes are efficient, but Lai et al.'s scheme is vulnerable to the privileged insider attack and off-line password guessing attack, while Lee et al.'s scheme is vulnerable to man-in-the-middle attack once after the adversary gets to know the identities of at least two users, which in practice, is feasible.

## Conclusion

In this paper, we proposed the first anonymous three-party password-authenticated key exchange scheme based on elliptic curve cryptosystem. Anonymity, authentication and security of the proposed scheme are validated using the applied pi calculus based formal verification tool ProVerif. The proposed scheme is secure and efficient, and is suitable for applications in telecare medical information systems.
